# Knowledge of medication dispensing among community pharmacists in Brazil: A national cross-sectional survey

**DOI:** 10.1371/journal.pone.0326817

**Published:** 2025-10-06

**Authors:** João Paulo Alves Cunha, João Paulo Vilela Rodrigues, Kérilin Stancini Santos Rocha, Ana Maria Rosa Freato Gonçalves, Fabiana Rossi Varallo, Leoanardo Régis Leira Pereira

**Affiliations:** 1 Pharmaceutical Assistance and Clinical Pharmacy Research Center, Department of Pharmaceutical Sciences, School of Pharmaceutical Sciences of Ribeirão Preto, University of São Paulo (USP), Ribeirão Preto, Brazil; 2 Laboratory for Innovation in Health Care, Graduate Program in Pharmaceutical Sciences, Federal University of Espírito Santo, Vitória, Brazil; University of Science and Technology of Fujairah, YEMEN

## Abstract

Brazilian community pharmacists’ knowledge of medication dispensing was assessed through a nationally representative cross-sectional survey conducted between October 2021 and May 2022. A total of 366 licensed pharmacists working in private community pharmacies—71.3% of whom were female, with an average age of 36.4 years (standard deviation 9.4)—completed a validated online questionnaire covering fundamental aspects of dispensing practice and reported on their undergraduate training and preferred information sources. The overall mean correct-response rate was 70.8%, with the Southeast and South regions scoring highest at 71.0% and 71.9%, respectively. Domains related to antimicrobial and over-the-counter medication dispensing exhibited the largest knowledge gaps across all regions. Graduates of public universities and pharmacists holding postgraduate qualifications demonstrated significantly greater proficiency, and higher knowledge scores correlated with more positive perceptions of the relevance of their training for patient counseling and community health promotion. Internet resources and medication package inserts were the most frequently consulted references for resolving dispensing questions, with 38% of respondents consulting them on a daily basis. These findings reveal a moderate overall level of dispensing competence, pinpoint critical areas for improvement, and underscore the influence of educational background on professional performance. Targeted continuing-education initiatives are therefore essential to reinforce pharmacists’ competencies, enhance the quality of services in community pharmacies, and ultimately improve patient care outcomes.

## Introduction

Community pharmacies are the most accessible healthcare facilities for the population and are, therefore, a favorable setting for the implementation of services that improve health outcomes and people’s quality of life. Pharmaceutical Care provided in a community pharmacy encompasses clinical services such as medication reconciliation, pharmacotherapeutic follow-up, pharmacotherapy review, monitoring of clinical parameters, and medication dispensing [1–4]. To conduct proper medication dispensing, which involves ensuring the provision of drugs through the analysis of the technical and legal aspects of prescriptions and actions that include pharmaceutical counseling and documentation of interventions [2,5], the presence of a pharmacist with clinical and humanistic training is essential. The quality of any pharmaceutical service is directly linked to the pharmacists’ academic education and practical training.

In the US, it is estimated that 57% of pharmacists work in establishments that perform dispensing [6]. In Brazil, the Federal Pharmacy Council, that regulates the pharmacy profession, indicates that approximately 70% of licensed pharmacists work in community pharmacies. Among them, about 85% practice in private establishments [7]. According to the European Community Pharmacists, the population aging observed in recent decades, and the increase in the prevalence of chronic diseases pose new challenges to health systems worldwide. In this context, the role of pharmacists in activities such as medication dispensing has great potential to promote adherence to drug therapies, improve health outcomes, and reduce the demand for care in higher-complexity health facilities and overall healthcare costs [1]. A systematic review conducted by Pizetta et al., 2021, showed that most of the included studies demonstrated a positive impact of dispensing on the clinical outcomes of different health conditions [8]. However, despite this recognized importance, studies evaluating the knowledge of community pharmacists regarding medication dispensing are scarce, especially in developing countries.

It is recommended that education for clinical practice be based on the development of competencies that encompass knowledge, skills, and attitudes through practical activities in real-life settings or real-life situations simulation [9,10]. The lack of such studies creates a barrier to a situational diagnosis and the planning of educational actions to address potential gaps. In this context, the study aimed to assess the knowledge of Brazilian pharmacists working in community pharmacies about medication dispensing, as well as to identify their perceptions of their academic training.

## Methods

### Study design and setting

This descriptive, cross-sectional survey was conducted online, reaching participants from all Brazilian Federal Units between October 27, 2021, and May 20, 2022. The participating pharmacists worked in community pharmacies located in predominantly urban settings. The study followed the recommendations of Kelley et al. (2003) and Bennett et al. (2011) and was reported according to the Consensus-Based Checklist for Reporting of Survey Studies (CROSS) [11,12].

### Study participants

All licensed pharmacists with active registration in their respective Regional Pharmacy Councils, working in private community pharmacies in any Brazilian Federal Unit and performing medication dispensing as part of their routine, were invited to participate. No exclusion criteria were applied.

### Sample size calculation

According to the Federal Pharmacy Council, Brazil had 234,301 registered pharmacists in 2020 [13]. A sample size of 337 pharmacists was calculated for a 5% margin of error and 95% confidence level, considering the finite population. To reach the required sample, a non-probabilistic approach using convenience and snowball sampling was employed [14]. Recruitment was initiated by disseminating the survey link through the researchers’ professional networks and social media platforms, with participants being encouraged to share it with eligible colleagues. While this method was practical for achieving broad geographic coverage, we acknowledge that it introduces a potential for selection bias and may limit the generalizability of the findings.

### Data collection instruments

The study was carried out by sending two previously validated structured questionnaires online.

1. Questionnaire for the Evaluation of Knowledge about Medication Dispensing (CDM-51) [15].

This questionnaire was developed and validated for Portuguese by Gonçalves and collaborators (2018) and consists of two independent sections. The first refers to the sociodemographic data and schooling of the research participants and has 10 items, while the second part has 51 objective questions, which can be answered with “Yes”, ‘No’ or “I don’t know” and aims to assess the participants’ knowledge about dispensing medicines in six dimensions: 1) Attitudes allowed in the pharmaceutical environment (10 items); 2) Dispensing medicines subject to special control (13 items); 3) Dispensing generic medicines (four items); 4) Dispensing antimicrobials (seven items); 5) Dispensing non-prescription medicines (four items); 6) Dispensing medicines belonging to the Farmácia Popular do Brasil program (government program for access to affordable medicines) and/or used (13 items). The original validation study reported good internal consistency for the instrument, with a Kuder-Richardson 20 (KR-20) value of 0.837 [15].

1. Instrument developed by Redigolo (2018) [16].

This instrument assesses the appreciation of training in the Pharmacy undergraduate course, and aims to identify the survey participants’ perception of the usefulness of their training during graduation in carrying out certain professional activities, including dispensing medicines. The instrument is made up of 20 items and uses a six-point Likert Scale to collect responses, with 0 being the lowest point on the scale representing that the participant had no training during graduation for the activity specified in the statement, and 5 being the highest point on the scale representing that the training received by the participant was extremely useful for carrying out the given activity.

For this study, a subset of 10 items was selected from the original 20-item instrument. This adaptation aimed to reduce participant burden and focus on activities most central to medication dispensing in the Brazilian community pharmacy context. The topics selected were: Filling in the Notification of Irregularity of Medicines suspected of quality deviation or adverse reaction; Delivering medicines; Advising on the availability of items; Informing on the validity of the prescription; Clarifying doubts and/or any other need; Clarifying doubts related to the use of medicines; Guidance on the use of gynecological products; Glycemic monitoring; Pharmacotherapeutic follow-up; General health promotion in the community. The internal consistency of this 10-item adapted scale was assessed in our sample, yielding a Cronbach’s alpha of 0.961.

### Research protocol

Initially, the research instruments were made available in electronic format via Google Forms® and submitted to a pilot study. At this stage, 20 pharmacists with previous experience in dispensing medicines at some point in their professional career took part. The participants answered the instruments in full and, at the end, were able to register their opinions and suggestions about the electronic form, addressing aspects such as layout, average response time, any grammatical errors and the clarity of the questions. Based on the feedback received, minor changes were made, without altering the construct of the instruments, and an average response time of 20 minutes was established.

After this stage, the instruments were disseminated remotely, still using Google Forms®, between October 2021 and May 2022, with the aim of reaching as many pharmacists working in private community pharmacies as possible. The survey was disseminated mainly through social networks (Facebook, Instagram and WhatsApp). Access to the questionnaire was made available both via a QR Code, inserted into graphic materials developed by the researcher to facilitate dissemination, and via a URL (Uniform Resource Locator) link incorporated into the text of posts and shared messages.

In addition, all the Regional Pharmacy Councils in Brazil, as well as the Federal Pharmacy Council, were contacted by telephone and/or e-mail to ask for support in disseminating the survey to pharmacists with active registration.

It should be noted that the questionnaire was anonymous and self-administered, with all the mandatory questions, guaranteeing complete answers. To prevent multiple submissions from the same individual, the Google Forms® settings were configured to limit one response per account. Furthermore, no financial incentives were offered for taking part in the study.

### Statistical analysis

Continuous variables were presented using mean, standard deviation, minimum and maximum values, while categorical variables were presented using frequency. The Statical Package for Social Sciences® (SPSS) Inc., version 21, 2012 program was used to carry out the statistical analyses. The Poisson model (for comparing the means of discrete variables), Fisher’s exact test (for assessing the association between categorical variables) and the ANOVA test (for comparing the score and the degree of usefulness of the degree between the regions) were used to evaluate the variables between the regions. The comparison of pharmacists’ knowledge between sociodemographic variables and perception of academic training was carried out using Student’s t-test and ANOVA test (for categorical independent variables) and Pearson’s correlation coefficient (for quantitative independent variables). A significance level of 5% was set. The results were considered statistically significant when p ≤ 0.05.

### Ethical aspects

The Research Ethics Committee of the Faculty of Pharmaceutical Sciences of Ribeirão Preto (FCFRP-USP) approved the study (CAAE: 34271520.3.0000.5403; Opinion No. 5.058.567).

Informed consent was obtained electronically prior to participants accessing the questionnaire. Participants were presented with an online consent form detailing the study’s objectives, procedures, potential risks, and confidentiality measures. Only those who actively selected the ‘I agree to participate’ option were granted access to the questionnaire. All participants were 18 years of age or older. No minors were involved in the study.

## Results

### Sample characterization

A total of 366 pharmacists were included, representing all Brazilian regions, with the majority from the Southeast (52.5%; n = 192) and Northeast (24.6%; n = 90). The sample was predominantly female (71.3%; n = 261), a trend observed across all regions. The national mean age was 36.4 ± 9.4 years (range: 22–70).

Table 1 presents participants’ academic backgrounds. The variables “Educational Qualification” (p = 0.01), “Years Since Pharmacy Graduation” (p < 0.001), and “Years of Professional Experience” (p < 0.001) differed significantly by region. Pharmacists in the South had the longest time since graduation, while those in the South and Southeast also reported the greatest dispensing experience.

**Table 1:** Educational profile of the pharmacists who participated in the study.

*Variables*	Brazil	North	Northeast	Southeast	South	Central-West
	*N = 366*	*N = 13*	*N = 90*	*N = 192*	*N = 55*	*N = 16*
**Pharmacists with previous education before their undergraduate degree N (%)**	12 (3.2)	2 (15.4)	1 (1.1)	7 (3.6)	2 (3.6)	0
Pharmacy Technician course	6 (50)	0	1 (100)	4 (57.1)	1 (50)	0
Pharmacy Assistant Course	6 (50)	2 (100)	0	3 (42.9)	1 (50)	0
**Qualification Type. N (%)**						
Generalist	264 (72.1)	10 (76.9)	81 (90)	122 (63.5)	37 (67.3)	14 (87.5)
Qualified in Industry	24 (6.6)	0	1 (1.1)	19 (9.9)	4 (7.3)	0
Qualified in Biochemistry	78 (21.3)	3 (23.1)	8 (8.9)	51 (26.6)	14 (25.5)	2 (12.5)
**Years Since Graduation. Mean (SD)**	10.8 (9.3)	6.8 (5)	6 (6.8)	12.6 (10.1)	13.7 (7.9)	9.3 (7.3)
**Type of Institution Attended for Undergraduate Degree. N (%)**						
Public	141 (38.5)	4 (30.8)	41 (45.6)	69 (35.9)	21 (38.2)	6 (37.5)
Private	225 (61.5)	9 (69.2)	49 (54.4)	123 (64.1)	34 (61.8)	10 (62.5)
Pharmacists with Postgraduate Education. N (%) *	266 (72.7)	10 (76.9)	61 (67.8)	137 (71.4)	45 (81.8)	13 (81.2)
Specialization in progress	52 (15)	2 (12.5)	19 (25.3)	22 (12.9)	6 (9.2)	3 (15.9)
Specialization completed	199 (57.5)	6 (37.5)	42 (56)	108 (63.2)	34 (52.3)	9 (47.4)
Improvement course in progress	5 (1.4)	0	0	1 (0.6)	3 (4.6)	1 (5.3)
Improvement course completed	29 (8.4)	2 (12.5)	4 (5.3)	13 (7.6)	9 (13.8)	1 (5.3)
Master’s degree in progress	11 (3.2)	0	2 (2.7)	6 (3.5)	2 (3.1)	1 (5.3)
Master’s degree completed	30 (8.7)	2 (12.5)	7 (9.3)	13 (7.6)	5 (7.7)	3 (15.9)
PhD’s degree in progress	6 (1.7)	2 (12.5)	0	2 (1.2)	1 (1.5)	1 (5.3)
PhD’s degree completed	14 (4)	2 (12.5)	1 (1.3)	6 (3.5)	5 (7.7)	0
**Pharmacists with Postgraduate Education in Dispensing. N (%)**	86 (23.5)	3 (23.1)	29 (32.3)	40 (20.8)	13 (23.6)	1 (6.3)
**Years of Experience in Dispensing. Mean (SD)**	10.9 (9.4)	6.1 (5.1)	6.6 (7.2)	12.6 (10.3)	13.9 (7.7)	9.4 (6.8)

Most pharmacists (97%; n = 355) reported having encountered questions about medications, with 100% of participants in the North confirming this. The most frequently used resources for clarification were the Internet (44.2%) and drug package inserts (21%), showing similar patterns across regions. Among those reporting doubts, 38% (n = 135) consulted these resources daily.

### Knowledge assessment

**Fig 1:**
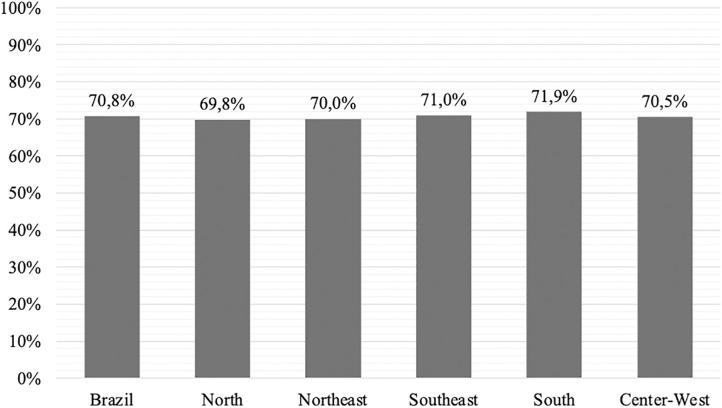
Regional and national averages of the percentage of correct answers on the CDM-51.

The national mean percentage of correct responses on the CDM-51 was 70.8%. National and regional means are depicted in Fig 1. No significant differences were observed between regions (p = 0.868).

When examining each CDM-51 dimension separately, all showed mean scores above 50%. Two regions exceeded the national overall score: the Southeast (71%) and the South (71.9%), the latter achieving the highest regional score. Table 2 details pharmacists’ percent-correct scores by region across the CDM-51 dimensions. No significant interaction was found between dimensions and region (p = 0.751).

**Table 2:** Regional and national averages of the percentage of correct answers in the domains comprising the CDM-51.

*Domains (CDM-51)*	Brazil	North	Northeast	Southeast	South	Central-West
	*N = 366*	*N = 13*	*N = 90*	*N = 192*	*N = 55*	*N = 16*
Permitted Attitudes in the Pharmaceutical Environment (D1)	68.9%	73.1%	67.8%	69.3%	68.0%	68.8%
Dispensing of Controlled Substances (D2)	72.1%	74.0%	69.1%	72.5%	76.1%	69.7%
Dispensing of Generic Medicines (D3)	78.0%	75.0%	76.7%	78.4%	78.6%	81.3%
Dispensing of Antimicrobials (D4)	67.0%	63.7%	69.8%	66.6%	65.5%	63.4%
Dispensing of Over-the-Counter Medicines (D5)	64.7%	63.5%	66.1%	63.9%	54.2%	56.3%
Dispensing of Medicines from the Brazilian Popular Pharmacy Program and/or Commonly Used Medicines (D6)	72.8%	66.9%	71.7%	73.2%	73.4%	77.4%

Correlations between CDM-51 scores and other study variables are presented in Table 3.

**Table 3:** Correlation between the average score on the CDM-51 and variables related to the training of community pharmacists.

*Variables*	Brazil (N = 366)	Mean *Score* CDM-51	*p*
Pharmacists with a course prior to their undergraduate degree. N (%)			
No	354 (96.8)	36.14	0.510
Yes. Pharmacy Technician Course	6 (1.6)	33.83	
Yes. Pharmacy Assistant Course	6 (1.6)	36.12	
**Type of Qualification. N (%)**			
Generalist	264 (72.1)	36.11	0.509
Qualified in Industry	24 (6.6)	37.33	
Qualified in Biochemistry	78 (21.3)	35.81	
**Type of Institution Attended for Undergraduate Degree. N (%)**			
Public	141 (38.5)	37.04	0.014*
Private	225 (61.5)	35.55	
**Pharmacists with Postgraduate Education. N (%)**			
Yes	266 (72.7)	36.98	0.001*
No	100 (27.3)	33.85	
**Pharmacists with Postgraduate Education in Dispensing. N (%)**			
Yes	86 (23.5)	37.52	0.01*
No	280 (76.5)	35.75	

*p < 0.05

### Perception of academic training

**Fig 2:**
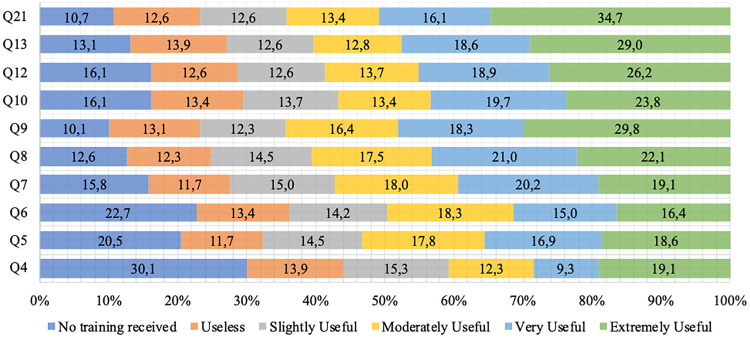
National sample’s perception of the usefulness of the pharmacy degree for performing activities related to medication dispensing. Q4 – Completion of Notification of Suspected Drug Quality Deviations or Adverse Reactions (e.g., “NOTIVISA”) Q5 – Delivery of medicines Q6 – Provide information on item availability Q7 – Inform about prescription validity Q8 – Clarify questions and/or any other needs Q9 – Clarify questions related to medication use Q10 – Guidance on the use of gynecological products Q12 – Blood glucose monitoring Q13 – Pharmacotherapeutic follow-up Q21 – General health promotion in the community.

Descriptive results regarding community pharmacists’ perceptions of their undergraduate training for medication-dispensing practices and related activities are shown in Fig 2.

Associations between CDM-51 scores and participants’ evaluations of the impact of their undergraduate education on dispensing activities are summarized in Table 4. Clarifying medication-use questions (Q9) and general health promotion in the community (Q21) were both significantly correlated with CDM-51 scores (p < 0.05). No other significant correlations were found between training perception and the sociodemographic or professional variables investigated (p > 0.05).

**Table 4:** Statistical analysis of possible correlations between the mean score on the CDM-51 and the perception of training for performing activities related to medication dispensing.

*Activities*	*p*
**Completion of Notification of Suspected Drug Quality Deviations or Adverse Reactions (e.g., “NOTIVISA”) (Q4)**	0.281
**Delivery of medicines (Q5)**	0.511
**Guidance on item availability (Q6)**	0.399
**Information about prescription validity (Q7)**	0.068
**Clarification of questions and/or any other needs (Q8)**	0.058
**Clarification of questions related to medication use (Q9)**	0.01*
**Guidance on the use of gynecological products (Q10)**	0.237
**Blood glucose monitoring (Q12)**	0.112
**Pharmacotherapeutic follow-up (Q13)**	0.224
**General health promotion in the community (Q21)**	0.027*

*p < 0.005

## Discussion

This is the first nationwide study to evaluate Brazilian community pharmacists’ knowledge of medication dispensing, a critical field of inquiry given that nearly 60% of the country’s registered pharmacists work in this setting [17]. Our main finding shows a mean knowledge score of 70.8%, which, although above a basic pass threshold, reveals substantial room for improvement. This is particularly concerning as it involves competencies legally required for professional practice and directly linked to patient safety [10,18]. The most significant knowledge deficits were observed in the domains of antimicrobial and over-the-counter (OTC) medicine dispensing. Importantly, because our sample included participants from all regions of Brazil, capturing the nation’s considerable socioeconomic disparities [19], we were able to demonstrate a clear association between knowledge and educational background. Pharmacists with postgraduate qualifications and those graduating from public universities achieved significantly higher scores. Taken together, these results highlight systemic deficiencies in both foundational training and continuing education among community pharmacists in Brazil.

The low performance in antimicrobial dispensing is particularly alarming in light of the global crisis of antimicrobial resistance, which caused an estimated 1.3 million deaths worldwide in 2019 [20]. While the contribution of pharmacists to antimicrobial stewardship is well recognized in hospital settings [21–24], community pharmacists remain the primary point of contact for most patients. Insufficient knowledge at the moment of dispensing undermines national initiatives to promote rational antimicrobial use, with consequences for clinical outcomes and healthcare costs [25–27].

Similarly, inadequate knowledge of OTC medicines represents a direct risk to patient safety. Consistent with the findings of Mota et al. (2020), our results confirm that patients frequently underestimate the risks of non-prescription drugs, including drug–drug interactions, contraindications, and adverse effects, particularly in vulnerable populations such as older adults, children, and pregnant women [28,29]. When pharmacists lack the knowledge to provide appropriate guidance, their critical role as the final safeguard in the medication-use process is compromised. This underscores the urgent need for targeted educational interventions.

Our results strongly suggest that the observed knowledge gaps are rooted in the quality and orientation of pharmaceutical education. The superior performance of pharmacists with postgraduate training—especially in clinical fields (p < 0.001)—underscores the effectiveness of specialized continuing education [30]. Equally concerning is the disparity between graduates of public (higher scores) and private universities (p = 0.014), given that private institutions account for the majority of pharmacy programs in Brazil [31]. This points to possible heterogeneity in curriculum quality, faculty expertise, and institutional infrastructure, as previously reported by Reis et al. (2015) and corroborated by national student performance assessments [31,32]. Such educational shortcomings likely contribute directly to suboptimal professional performance after graduation [33,34].

Addressing these deficiencies requires multifaceted solutions. Identifying gaps alone is insufficient; proactive measures must be implemented. Drawing on successful initiatives, such as the training in medication dispensing offered by the Brazilian Ministry of Health to public-sector pharmacists between 2019 and 2024 [35], we recommend expanding such strategies to the private sector. Furthermore, establishing national continuing education programs, potentially mandated for professional license renewal, and strengthening partnerships between academic institutions and community pharmacies could help bridge the theory–practice divide, offering students real-world training opportunities through active learning methodologies [10].

Another critical finding was pharmacists’ reliance on low-quality information sources in daily practice [36–40]. This behavior perpetuates knowledge gaps and endangers patients. It is therefore essential to promote the use of accessible, evidence-based resources. Pharmacists should be trained and encouraged to rely on authoritative sources such as national formularies (e.g., *Formulário Terapêutico Nacional* – FORMUN) and guidelines issued by the World Health Organization (WHO) and the Pan American Health Organization (PAHO). Barriers to their use—whether lack of awareness, subscription costs, or difficulties integrating them into routine workflow—should be systematically investigated and addressed by professional organizations.

This study has limitations. The use of non-probabilistic sampling (convenience and snowball) may introduce selection bias, potentially limiting the generalizability of our findings to all community pharmacists in Brazil. In addition, as the survey was self-administered, social desirability bias cannot be entirely ruled out. Nevertheless, this study provides an unprecedented national overview of pharmacists’ knowledge in medication dispensing, identifying urgent priorities for educational reform and professional development. Future research should also strengthen psychometric validation, for instance, through test–retest reliability analysis.

## Conclusion

The mean correct-response rate in the knowledge assessment of Brazilian pharmacists regarding medication dispensing was 70.8%. Training at public institutions and completion of postgraduate education were associated with higher knowledge levels. Considering that dispensing is one of the primary services provided to the public in community pharmacies, it is essential to implement strategies—including continuing education—to strengthen pharmacists’ qualifications and, consequently, improve the quality and effectiveness of the service delivered.

## Supporting information

S1 File Database. (XLSX)
